# Protocol for a cluster randomised trial in Madhya Pradesh, India: community health promotion and medical provision and impact on neonates (CHAMPION2); and support to rural India’s public education system and impact on numeracy and literacy scores (STRIPES2)

**DOI:** 10.1186/s13063-020-04339-6

**Published:** 2020-06-25

**Authors:** Arjun Agarwal, Rukmini Banerji, Peter Boone, Diana Elbourne, Ila Fazzio, Chris Frost, Madan Gopal, Sridevi Karnati, Rakhi Nair, Harshavardhan Reddy, Padmanabh Reddy, Dropti Sharma, Sajjan Singh Shekhawat, Siddharudha Shivalli

**Affiliations:** 1 Pratham Education Foundation, New Delhi, India; 2 Effective Intervention, London, UK; 3 London School of Hygiene and Tropical Medicine, London, UK; 4 NICE Foundation, Hyderabad, India; 5 GH Training and Consulting, Hyderabad, India

**Keywords:** Cluster randomised controlled trial, India, Neonatal mortality, Immediate neonatal care, Postnatal care, Maternal mortality, Stillbirths, Primary education, Supplementary teaching, Literacy, Numeracy

## Abstract

**Background:**

Rural areas of India exhibit high neonatal mortality, and low literacy and numeracy. We assess the effect of a complex package of health interventions on neonatal survival and the effect of out-of-school-hours teaching on children’s literacy and numeracy in rural Madhya Pradesh.

**Methods/design:**

This is a cluster-randomised controlled trial with villages (clusters) receiving either a health (CHAMPION2) or education (STRIPES2) intervention. Building on the design of the earlier CHAMPION/STRIPES trial, villages receiving the health intervention are controls for the education intervention and vice versa.

The clusters are 196 villages in Satna district, Madhya Pradesh, India: each is at least 5 km from a Community Health Centre, has a population below 2500, and has at least 15 children eligible for the education intervention. The participants in CHAMPION2 are resident married women younger than 50 years of age who had not undergone a family planning operation, provided they are enumerated pre-randomisation or marry a man enumerated pre-randomisation. The participants in STRIPES2 are resident children born 16 June 2010 to 15 June 2013, not in school before the 2018–2019 school year and intending to enrol in first grade in 2018–2019 or 2019–2020.

**Discussion:**

In CHAMPION2, the NICE Foundation will deliver a 3.5-year programme comprising Accredited Social Health Activists or village health workers and midwives promoting health knowledge and providing antenatal, postnatal, and neonatal healthcare; community mobilisation; referrals to appropriate government health facilities; and a health education campaign. In STRIPES2, the Pratham Education Foundation will deliver a programme of village-based, before/after school support focusing on literacy and numeracy. As controls, the CHAMPION2 control villages will receive the usual health services (plus the STRIPES2 intervention). STRIPES2 control villages will receive the usual education services (plus the CHAMPION2 intervention). The primary outcome in CHAMPION2 is neonatal mortality. Secondary outcomes include antenatal, delivery, immediate neonatal and postnatal care practices, maternal mortality, stillbirths, early neonatal deaths, perinatal deaths, health knowledge, hospital admissions, maternal blood transfusions, and cost effectiveness. The primary outcome in STRIPES2 is a composite literacy and numeracy test score. Secondary outcomes include separate literacy and numeracy scores, reported school enrolment and attendance, parents’ engagement with children’s learning, and cost effectiveness. Independent research and implementation teams will conduct the trial. Trial Steering and Data Monitoring Committees, with independent members, will supervise the trial.

**Trial registration:**

Clinical Trial Registry of India: CTRI/2019/05/019296. Registered on 23 May 2019. http://www.ctri.nic.in/Clinicaltrials/pdf_generate.php?trialid=31198&EncHid=&modid=&compid=%27,%2731198det%27

**Administrative information**

Note: the numbers in curly brackets in this protocol refer to SPIRIT checklist item numbers. The order of the items has been modified to group similar items (see http://www.equator-network.org/reporting-guidelines/spirit-2013-statement-defining-standard-protocol-items-for-clinical-trials/).

**Table:** 

Title {1}	Protocol for a cluster randomised trial in Madhya Pradesh, India: Community health promotion and medical provision and impact on neonates (CHAMPION2); and support to rural India’s public education system and impact on numeracy and literacy scores (STRIPES2)
Trial registration {2a and 2b}.	This trial is registered in the Clinical Trial Registry of India (CTRI/2019/05/019296)
Protocol version {3}	20th March 2020 MP trial protocol 11th Version
Funding {4}	Effective Intervention NGO
Author details {5a}	Arjun Agarwal (AA), Pratham Education Foundation Rukmini Banerji (RB), Pratham Education Foundation Peter Boone (PB), Effective Intervention Diana Elbourne (DE), London School of Hygiene and Tropical Medicine Ila Fazzio (IF), Effective Intervention Chris Frost (CF), London School of Hygiene and Tropical Medicine Madan Gopal (MG), NICE Foundation Sridevi Karnati (SK), GH Training and Consulting Rakhi Nair (RN), NICE Foundation Harshavardhan Reddy (HR), GH Training and Consulting Padmanabh Reddy (PR), NICE Foundation Dropti Sharma (DS), Pratham Education Foundation Sajjan Singh Shekhawat (SS), Pratham Education Foundation Siddharudha Shivalli (SiS), London School of Hygiene and Tropical Medicine
Name and contact information for the trial sponsor {5b}	Effective Intervention, Centre for Economic Performance, London School of Economics, UK. Email: admin@effint.org
Role of sponsor {5c}	The research manager of Effective Intervention has participated in the study design and writing of the protocol

## Introduction

### Background and rationale {6a}

#### CHAMPION2

It is estimated that 2.6 million neonatal deaths occur annually, of which 24% (640,000) are in India [1]. In 2015, the major determinants of neonatal deaths in India were prematurity and low birth weight (44%), birth asphyxia and birth trauma (19%), and neonatal infections (19%) [2].

Despite India’s rapid economic growth, there has been little progress in improving neonatal survival [2]. According to the Sample Registration System Statistical Report 2017, India’s neonatal mortality rate (NMR) is 23 per 1000 live births [3]**.** Estimates vary widely between the different states i.e. the NMR is 5 per 1000 live births in Kerala and 33 per 1000 live births in Madhya Pradesh [3]. There are large disparities in health, with NMRs almost twice higher in rural areas compared to urban areas (14 and 27 per 1000 live births in urban and rural areas, respectively) [3]**.** The state of Madhya Pradesh is characterised by a marginalised, tribal population, where less than 30% of mothers in rural villages have four or more antenatal care visits and the proportion of illiterate women is about 50% [4]. Within the state, the NMR ranges between 24 per 1000 live births in Indore district, and 57 per 1000 live births in Satna district [5].

India, like other South Asian countries, has national programmes aimed at improving maternal and neonatal health. In 2005, India’s National Rural Health Mission (NRHM), now a component of the National Health Mission, launched *Janani Suraksha Yojana* (JSY), a major safe motherhood intervention to reduce maternal and neonatal mortality by promoting institutional deliveries [6]. JSY is a conditional cash transfer scheme targeting poor pregnant women who deliver in health facilities and receive postnatal care [6].

The JSY is responsible for increasing the proportion of deliveries occurring in health facilities from 26% in 2005 to 81% in 2015 in Madhya Pradesh [4, 6]. Despite this initiative, there has not been a corresponding reduction in neonatal mortality in Madhya Pradesh [7]. There is also evidence suggesting that the quality of care in some facilities is substandard [7, 8].

Another key element of the NRHM is the Accredited Social Health Activist (ASHA). The ASHA acts as an effective link between the government and community by facilitating pregnant women in accessing antenatal care, delivery in a health facility, postnatal care, and other available health services. The ASHA receives performance-based incentives for her various services, which include referral and escort services for pregnant women under NRHM [6, 9]. ASHAs have also been trained to provide home-based neonatal care through postnatal home visits and providing health education through community mobilisation [9]. Evaluations have shown that the NRHM has had a positive impact on increasing attendance at antenatal care and institutional deliveries, and on immunisation rates [8].

Scaling up community programmes worldwide to improve neonatal health has the potential to reduce neonatal mortality by 24% [10]. Community support, community mobilisation, home visits by trained community health workers, and strengthening referral systems are the most effective community-based interventions [10]. Evidence-based research has also prompted the World Health Organisation (WHO) and UNICEF’s Every Newborn Action Plan to recommend these approaches [11].

Over the past 15 years, there have been several trials aiming to improve maternal and neonatal health through home visits; some of these trials took place in India [12–14]. A study in Uttar Pradesh, India, found that an intervention that included home visits and community meetings led to improved care practices and neonatal survival [13]. Another study evaluated the effectiveness of scaling up India’s Integrated Management of Neonatal and Childhood Illness (IMNCI) strategy that included postnatal home visits and treating sick neonates in accordance to pre-defined guidelines. Findings suggested that the IMNCI strategy has the potential to reduce neonatal mortality by 12% when brought to scale [14].

Community mobilisation is the other main approach to community health, in particular, community mobilisation through participatory learning and action (PLA) [15]. PLA includes women’s groups operated by women identifying problems relevant to their own health needs, coming up with culturally acceptable solutions, and implementing these solutions [15]. A meta-analysis and systematic review testing the impact of community mobilisation using PLA with women’s groups, which also included five studies from South Asia, suggested this to be a highly effective and low-cost intervention in reducing neonatal and maternal mortality [15]. A recent cluster randomised controlled trial (cRCT) demonstrated that ASHAs can reduce neonatal mortality using the PLA approach with women’s groups [16]. The Indian government has now mandated ASHAs to deliver PLA through women’s groups in 18 separate states in India, beginning with Odisha and Jharkhand [17].

The original CHAMPION trial randomised 464 villages in Telangana, India. Villages were randomised to an intervention aimed at reducing neonatal mortality or to a control arm offering the usual ongoing health services. The health intervention included a package comprising community health promotion (i.e. health education through village health worker-led participatory discussion groups), outreach (i.e. mobile teams providing antenatal and postnatal care in the home or through fixed day health services), and provision of facility-based care (i.e. subsidised access to non-public health centres) [18]. The primary outcome of neonatal mortality was significantly lower in the intervention arm compared to the control arm (52 neonatal deaths per 1000 live births versus 69 deaths per 1000 live births), a reduction of 24% (relative risk 0.76; 95% CI 0.64 to 0.90; *p* = 0.0018). The authors concluded that the CHAMPION intervention was strongly justified in this setting, but the trial needed adaptation and further development for evaluation in other areas [18].

The objective of the CHAMPION2 trial is to assess whether an adapted intervention is also able to improve neonatal survival, albeit in a different region of India. The original study design will be further developed using lessons learned from the original CHAMPION trial, as well as relevant evidence generated since the conception of the original trial. The CHAMPION2 trial will also be contextualised to socio-cultural patterns and behaviours relating to neonatal and maternal health in Madhya Pradesh, India.

#### STRIPES2

India has made steady progress in improving rates of primary school enrolment. In rural areas, about 97% of children between 6 and 14 years of age are now in school [19]. The levels of learning achievement, however, remain low. The 2018 Annual Status of Education Report (ASER) survey shows that proficiency in reading and numeracy is worryingly low and Indian children may spend several years in school without learning even the basic skills in literacy and numeracy [19].

Several strategies have been proposed to address the low quality of education, but many of these approaches fail to improve children’s learning as they tend to exclusively address the provision of facilities, materials and access to school rather than improve the child’s experience in the learning process [20–25].

Few studies have systematically measured the impact of supplementary teaching on learning outcomes. Evidence from Chile and India shows that providing extra teaching with tutors hired from the local community successfully raises the scores of low-performing children [26–28]. A 3-month tutoring programme targeting fourth grade, low-performing students in Chile significantly increased pupils’ reading skills [28]. In India, a programme that provided remedial classes for third and fourth grade pupils in public schools of Mumbai and Vadodara increased the overall test scores in literacy and numeracy by 0.28 SD in the second year of the programme [26]. Another study in Uttar Pradesh, India showed a positive impact on reading skills in children who had a high attendance in reading camps led by community volunteers [25].

In Telangana, the STRIPES trial intervention, which included children attending public primary schools, demonstrated important results in improving numeracy and language scores [27]. This trial evaluated the effectiveness of an intervention that provided 18 months supplementary, remedial teaching and learning materials (and an additional “kit” of materials for girls). The primary outcome was a composite of language and numeracy test scores. These scores were significantly higher in the intervention group (107 villages; 2364 children) than in the control group (106 villages; 2014 children) at the end of the trial (mean difference on a percentage scale 15.8; 95% CI 13.1 to 18.6; *p* = 0.001; 0.75 Standard Deviation (SD) difference). Composite test scores were not significantly different in the 54 villages (614 girls) with the additional kits for girls compared to the 53 villages (636 girls) without these kits at the end of the trial (mean difference on a percentage scale 0.5; 95% CI − 4.34 to 5.4; *p* = 0.84). The STRIPES trial provided strong evidence that supplementary teaching implemented in remote rural areas has a strong impact in improving numeracy and literacy skills. Given the little evidence found in previous trials, it would be beneficial to adapt and further develop the STRIPES trial to determine whether the study findings are generalizable to different settings.

Taking into consideration the above evidence, the primary aim of STRIPES2 is to assess whether an intervention adapted from Pratham’s current model of working with early grades has a similar effect on the literacy and numeracy of primary school age children in Madhya Pradesh, using a design developed from the STRIPES trial.

## Objectives {7}

Our main research question is whether the success of the CHAMPION and STRIPES trial interventions can be further generalised, after appropriate adaptations and developments, in Madhya Pradesh. This new cluster-randomised trial therefore combines the further developed versions of the previous CHAMPION (addressing neonatal survival) and STRIPES (addressing learning levels among primary-school-age children) interventions. Participants in the control arm of the health intervention will receive the education intervention, whereas participants in the control arm of the education intervention will receive the health intervention.

This protocol has been written taking into account the Standard Protocol Items: Recommendation for Interventional Trials (SPIRIT) guidelines [29].

## Trial design {8}

This study is a cluster-randomised controlled trial with villages receiving either a health (CHAMPION2) or education (STRIPES2) intervention.

## Methods: participants, interventions and outcomes

### Study setting {9}

The trial will take place in Satna district, Madhya Pradesh, with a population of 2.23 million, of which 79% live in a rural setting with a predominantly agrarian economy [30, 31]. In terms of education, between 2006 and 2018, Madhya Pradesh has experienced an increase in school enrolment rates [19]. In rural villages, 96% of children between 6 and 14 years old, and 94% between 11 and 14 years old were enrolled in school in 2018 [19]. However, the achievement in basic reading was very low with only about 18% of children in grade 3 being able to read a short story (7–10 sentences) in the local language [19].

In terms of neonatal health, Satna has one of the highest NMRs in Madhya Pradesh, with a higher NMR in rural compared to urban areas (61 per 1000 live births in rural areas, and 43 per 1000 live births in urban areas) [5]. Satna is also the second worst performing district in terms of neonatal, infant, and child mortality in Madhya Pradesh [4], which is the state with the highest NMR in India [3].

The Government delivers health care through Sub-Centres, Primary Health Centres (PHCs), Community Health Centres (CHCs) and Civil Hospitals (CHs), and one District Hospital (DH), which has a Special Newborn Care Unit (SNCU).

In Satna district, only 23.1% of the pregnant women had four or more antenatal care visits, 85.8% received tetanus toxoid vaccination, 17.1% consumed iron folic acid (IFA) for 100 days or more, and 7.6% received full antenatal care (i.e. at least four or more antenatal care visits, at least one tetanus toxoid injection, and IFA for 100 days or more) [4]. Overall, 80% of deliveries in the district occur in health facilities [4]. Approximately 56.2% of mothers received a postnatal check-up within 48 h of delivery [4]. Satna district has an ambulance system (the 108 and Janani Surakhsa Express (JE)), responsible for transporting pregnant women and babies with serious health complications to the district hospital [32]. Whereas JE was considered relatively accessible to even the most marginalised populations, anecdotal evidence suggests that in practice there may be long delays, especially in areas that are difficult to access [32, 33]. Recently these two systems have been merged, and a single transport system (108) addresses all kinds of medical needs, not just pregnancy and neonatal needs. Hence, transport may not always be speedily available.

Emergency obstetric care (EmOC) is a package of medical interventions required to treat seven obstetric complications [34, 35]. The medical interventions included in this package comprise parenteral antibiotics, oxytocic drugs and anticonvulsants, manual removal of placenta, removal of retained products, and assisted vaginal deliveries [35]. Evidence suggests that birth attendants in JSY facilities in Madhya Pradesh are often not able to perform EmOC adequately [6, 36]. These findings may help to explain why there has not been a corresponding improvement in neonatal survival despite increased rates of facility-based deliveries.

### Eligibility criteria {10}

The trial will be conducted in Satna district, Madhya Pradesh, India. Satna district is further divided into 10 *tehsils* (sub-districts). Three *tehsils* (Birsinghpur, Majhgawan, and Raghurajnagar) were excluded due to difficult access (forest area), risk of violent robbery, and being an urban sub-district. The remaining seven *tehsils* potentially eligible for the trial comprise 1263 villages (68% of all villages in Satna), with a population of 1,441,930 [30].

In these seven *tehsils*, villages were included if they:

1. Were considered rural, with fewer than 2500 population and with more than 120 children under the age of 6 years [30] (villages where we did not find at least 15 children eligible for STRIPES2 were excluded after enumeration);
2. Were accessible by road;
3. Were not within a 5 km radius of the Community Health Centres (as such villages are already well-served by the local health services);
4. Had a minimum of 3 km between the centres of the villages (to avoid “contamination”, though even with that buffer we may have some contamination as villages are very spread out).

From July 2017 to January 2018, we conducted a baseline enumeration to enlist the eligible women and children.

A woman was eligible for CHAMPION2 if during enumeration she satisfied all the following criteria:

1. She was married
2. Neither she nor her husband had a family planning operation (i.e. tubectomy or vasectomy)
3. She was younger than 50 years of age
4. She was resident of one of the trial villages at the time of the baseline survey
5. She gave her consent after being given a complete explanation of the study

In addition, a woman was considered eligible if she married a man who was enumerated and unmarried at the time of enumeration, resident of the village, and aged between 13 and 50 years. If an eligible woman died, her widowed husband was added to the list of unmarried men. If this widowed man married again then his wife was considered eligible.

The woman had to fulfil the usual criteria for eligibility including being younger than 50 years of age, resident of the village, gave her consent, and neither she nor her husband have had a family planning operation. Women (and their babies) who moved to a trial village for other reasons (e.g. their mother’s residence for delivery of their baby) were not included in the study, and will not have access to the antenatal and postnatal care provided by the intervention team (unless they were enumerated in another CHAMPION2 intervention village).

For the primary trial analysis, we will start counting births and deaths 12 months after the randomisation. This lag in measurement is necessary to ensure adequate exposure to the intervention taking into account both the time interval between conception and birth, and the fact that training and establishment of services in intervention villages will take around 6 months.

A child was considered eligible for STRIPES2 if:

1. She/he was born between 16 June 2010 and 15 June 2013^1^
2. She/he was not yet enrolled in primary school
3. She/he was expected to be resident in the village and be enrolled in school for the first time, in the first grade in the academic year of 2018–2019
4. Her/his parents agreed that she/he participates after hearing the explanation about the programme and its evaluation tests

For STRIPES2, only children who are enumerated at baseline and present for the test will be considered in the final analysis. Children who move into the trial villages will not be included after enumeration.

Women and the parents of the children enumerated were informed that they may withdraw from the trial at any time.

Catch-up enumeration: before randomisation of villages, from April–June 2019 we conducted a catch-up enumeration in all the selected villages to enlist the eligible women and children who were missed during baseline enumeration, and updated the marital status of any previously enlisted unmarried men. If a man who was unmarried during baseline enumeration was discovered to have since married, then his name was removed from the unmarried men list and his wife was considered as eligible.

A child was considered eligible for STRIPES2 at the catch-up enumeration if:

1. She/he was born between 16 June 2010 and 15 June 2013^3^
2. She/he was enrolled in grade 1 in primary school in the 2018–19 school year or was planning to enter grade 1 in 2019–20
3. She/he was expected to be resident in the village in 2019–2020
4. Her/his parents agreed that she/he participates after hearing the explanation about the programme and its evaluation tests

Knowledge, attitudes, and practices (KAP) survey: before randomisation of villages, from November 2018 to January 2019, we conducted a KAP survey in 50 randomly selected villages, on women who had delivered a live baby within 2 years of the interview. We assessed health service utilisation, beneficial and harmful behaviours, and knowledge. This KAP survey will guide the need and topics of further formative research for the CHAMPION2 intervention.

### Who will take informed consent? {26a}

All women and the main person responsible for children who have been enumerated were informed about the trial and gave their consent to participate. During the trial, all women who are eligible to participate will be informed and asked to give consent.

### Additional consent provisions for collection and use of participant data and biological specimens {26b}

Participants may withdraw their consent at any time during the trial. No biological specimen will be collected during the trial. Women and caregivers will be free to accept or not any interview during the trial.

## Interventions

### Explanation for the choice of comparators {6b}

The comparator for the maternal and neonatal health package CHAMPION2 is the usual maternal and neonatal health for the area, as that is the locally accepted best practice. These villages will also receive the educational intervention STRIPES2, as this allows the evaluation of two independent interventions within one trial. Analogously, the comparator for the education package STRIPES2 is the usual education received in the area, as that is the locally accepted best practice. These villages will also receive the maternal and neonatal health intervention CHAMPION2.

### Intervention description {11a}

#### CHAMPION2

Formative research (quantitative and/or qualitative) may be carried out to help identify key areas (e.g. knowledge, attitudes, behaviour, care, etc.) in need of improvement through interviews and focus groups with local women, healthcare providers and village elders. The intervention in the CHAMPION2 trial will build on the knowledge acquired in the original CHAMPION trial as well as evidence published since the inception of the original trial. The intervention will work with the existing healthcare infrastructure and government services including *Anganwadis*, NICE Nurse Midwives (NMW) and ASHAs who will be tracking women and their babies in the antenatal period, at birth, and in the postnatal period. New services aimed at improving health knowledge and increasing the uptake of services will be created using ASHAs, Village Health Worker (VHWs) (recruited as part of the intervention when required as described below), and midwives. These community health workers will endeavour to ensure uniformity in quality of care across services in the intervention arm.

A survey on the services and related infrastructure of the health facilities was conducted according to the Indian Public Health Standards (IPHS) [37]. Based on this survey, NICE may facilitate modest improvements to services provided at some CHCs. A small percentage of high risk or emergency cases will be referred to tertiary-level care.

The place of delivery will be discussed during birth planning sessions and the intervention team will direct pregnant women to plan for deliveries at designated CHCs, CHs, or other hospitals. However, the final choice of where they wish to deliver will be with the family. Transportation to health facilities is currently provided by the government (108 ambulance). If government transport services are not available, the intervention team may arrange alternative transport for urgent cases. The intervention will include key elements as subsequently described.

#### Health promotion

A health awareness campaign will be launched in the different communities to promote knowledge relating to maternal and neonatal health. Examples of the different promotional strategies include focus groups and *Nukkad Natak’s* (village-level street plays). These will be adapted based on local customs to convey important messages to communities. There will also be discussion groups surrounding any concerns the communities have about the intervention. All members of the community, and not just women eligible for the trial, will be the focus of these campaigns.

#### Community mobilisation with women’s groups

Community mobilisation with women’s groups used in previous trials will be adapted to suit the context of the local population [15]. All the women of the village, not just the women eligible for the trial, will be allowed to participate. The ASHA (VHW) with PLA teams will lead the groups for sessions involving issues related to maternal and neonatal health. Discussions about the risk factors in pregnancy, the delivery, and the postnatal period will be facilitated, and solutions will be derived to tackle these issues. The objective of these meetings is to improve mothers’ health knowledge, encourage greater use of the fixed day service (see below), promote safe delivery, encourage delivery of high-risk pregnancies at an appropriate health centre, and to provide women a forum to discuss solutions to important issues in maternal and neonatal health.

#### Fixed day services (FDS) provided by ASHAs, VHWs, and NICE nurse midwives

Teams of two midwives will visit the intervention villages approximately every 2 weeks to offer a package of antenatal and postnatal care and other services relating to the mother and her baby. The midwives will offer these services after the women’s group session, assisted by an ASHA (VHW) based in the village. If a woman is unable to attend the services, the midwives and ASHA (VHW) will visit her at home. These services will be given only to pregnant women identified by NICE who have been enumerated and confirmed to be eligible for the trial.

Examples of the different components of antenatal care include the following: checking for anaemia (haemoglobin levels), weight, monitoring for pre-eclampsia (blood pressure, protein in urine), and monitoring for infections and gestational diabetes mellitus. Examples of services provided in the delivery period include the following: promotion of a clean delivery and other essential neonatal care practices, assisting a delivery where access to a health facility is not possible. Postnatal services can include the following: advice on breastfeeding (i.e. the importance of colostrum, initiating breastfeeding within an hour of delivery, exclusive breastfeeding for 6 months), care of the neonate (appropriate thermal care), and how to recognise danger signs in mothers and babies.

To access these services, pregnant women will receive the NICE ANC card for tracking immunisations, participation in health groups, hospitalisations, and regular check-ups (antenatal, postnatal, and neonatal). The midwives will keep a record of visits to the village, women seen, emergency deliveries assisted, and cases referred. The ASHAs (VHWs) and midwives will also keep a detailed record of all their cases and meet regularly to share information on their work and any problems encountered.

#### Referrals

ASHAs (VHWs), and midwives will be responsible for facilitating and monitoring the referrals of mothers and neonates to the nearest CHC or CH that has adequate human resources, equipment, medicines, and disposables available.

#### ASHAs (community health workers)

At the community level, ASHAs will have a critical role in delivering the intervention. A VHW may be appointed by the trial when the ASHA is not consistently available, not resident in the village, working in both intervention and control villages, not supporting the trial, or not performing well. The trial team will recruit VHWs from the local community based on their literacy, communication proficiency, previous knowledge and experience in pregnancy and childbirth (usually she will be from the same village, educated until at least the 5th grade, married, previously given birth, and recommended by the community).

The ASHAs (VHWs) will receive a combination of theoretical and practical training by midwives and physicians, on the following topics: monitoring pregnancies, identification of risk factors in both the expectant woman and their baby, clean delivery practices, appropriate thermal care, breast feeding, appropriate care-seeking, arranging the logistics of referrals in case of emergencies, building trust, and case management.

#### NICE nurse midwife

A Nurse Midwife (NMW) employed by NICE will provide the antenatal and postnatal care services after the women’s group discussions. They will also offer guidance to supervise ASHAs (VHWs). Midwives may conduct emergency deliveries if they occur during their visits to the villages and monitor referrals to health facilities. In the case of complications, the NMW will be responsible for referring the mother or neonate in distress to the next level of care. Midwives will be trained by physicians to deliver clear information related to pregnancy, childbirth, and neonatal care, and the use of communication tools to address deeply rooted superstitions and practices that may have negative impacts on the health of both mother and neonate.

### STRIPES2

The education intervention comprises before/after school teaching lessons for 2 h, typically 6 days a week given by a “Pratham Instructor” (PI). Each PI will be trained and paid to conduct teaching and learning activities in Hindi for a group of up to about 30 eligible children in the village. Thus, for the period of about 17 months the same cohort of children would receive the intervention. Pratham Cluster Leaders (CLs) will support, coordinate, and monitor the PI’s work.

The initial 2–3 weeks will be a warm-up phase. During this period, the PIs will carry out a series of activities, such as playing, singing, colouring, and drawing, to enable children to interact comfortably with the instructors. Thereafter, activities will be conducted to help PIs understand what children can do easily and what they are struggling with. After this period, the PI will begin the instructional classes. The subject matter covered in these sessions will reinforce the curriculum covered in school and will develop children’s literacy and numeracy skills in pace with their cognitive abilities.

Pratham has seen promising results when efforts are made to engage with the primary caregivers (usually mothers) of young children, to demonstrate and orient them on activities that they can do with their children at home [38]. PIs will thus have frequent contact with mothers through door-to-door meetings, community events, and creation of mothers’ groups. Mothers will be oriented to engage in activities with their children to promote their attendance and learning. The activities in communities will thus build on Pratham’s past experience; not only building a strong foundation, but also trying to create a sustainable learning environment that supports children’s development.

#### Material kits

Pratham will develop material kits to be used during the before/after school activities and at home. The material for the classes will include the instructor’s manual and teaching learning material such as story-books, activity booklets, and more. In addition, Pratham teams will orient mothers on use of the home materials. Orientations may be done in groups for easy comprehension of mothers or one by one. Videos may also be shown for this purpose.

### Community consultants

Conflicts have arisen in previous community trials with women who are dissatisfied with the services received from the intervention team (e.g. unfavourable pregnancy outcomes and expectation mismatch) or due to their ineligibility for the antenatal care (ANC) and postnatal care (PNC) services. One or more consultants with expertise in handling such conflicts will facilitate discussions to resolve these issues. A consultant will also train and support ASHAs (VHWs) and supervisors in interpersonal communication for effective interaction with the communities.

### Criteria for discontinuing or modifying allocated interventions {11b}

Although the PLA may be slightly modified according to local requests, there are no special criteria for discontinuing or modifying allocated interventions.

### Strategies to improve adherence to interventions {11c}

During the trial, the NICE and Pratham Foundations will regularly visit the households of the participants to promote awareness in a simplified manner. NICE nurses and ASHAs (or VHWs) will sensitise mothers to: keep track of their menstrual period, attend the PLA sessions and FDS, and remind pregnant women about the importance of their health assessments. Pratham instructors will regularly visit the households to engage families to send their children to the Pratham classes and explain to the caregivers how they can and should talk with their children in a playful way about topics that involve literacy and numeracy. Adherence will be monitored by both groups, daily in Pratham classes (pupils’ attendance) and every 2 weeks in the FDS (visits by enumerated pregnant women) and monthly in the PLA sessions (presence of enumerated women).

### Relevant concomitant care permitted or prohibited during the trial {11d}

The implementation of both Pratham classes and NICE services will not affect the usual school hours or the provision of antenatal and postnatal care that is normally provided by the government, *Anganwadis*, and ASHAs. Schools will function normally, and the health services provided by the government will be offered routinely to all women.

### Provisions for post-trial care {30}

There are no special provisions for ancillary care. Although there are no expectations of harm from trial participation, in the event of harms, appropriate compensation will be provided. The relevant interventions will be expanded to the controls after the trial if proven to be successful.

## Outcomes {12}

### CHAMPION2

The primary outcome in CHAMPION2 is neonatal mortality [39]. The secondary outcomes will include maternal mortality [39]; stillbirths and perinatal deaths [39]; causes of death [39]; antenatal care; delivery care; immediate neonatal care; postnatal care; health knowledge; hospital admissions of enrolled women during pregnancy or afterwards, or of their babies (during the neonatal period); maternal blood transfusions; and the cost effectiveness of the intervention.

### STRIPES2

The primary outcome of STRIPES2 is the composite literacy and numeracy test score using the Early Grade Reading Assessment (EGRA) and the Early Grade Mathematics Assessment (EGMA), respectively [40–42].

Secondary outcomes include separate scores for literacy and numeracy; parents’ engagement in their child’s learning; enrolment in school; parent’s report of school attendance; and the cost effectiveness of the intervention.

### Participant timeline {13}

#### Duration

Randomisation took place in June 2019. STRIPES2 will run for about 17 months and CHAMPION2 will run for 3½ years.

A timeline with the key events during the trial is presented in Table 1.

**Table 1:** Timeline of the key events in the trial

Date	Event
December 2015	Protocol submitted to the L V PRASAD EYE Institute (LVPEI) and London School of Hygiene and Tropical Medicine (LSHTM) Ethics Committees
January to October 2016	Permits sought and obtained from the Departments of Health and Education in Madhya Pradesh (Mission Director from the National Health Mission, Director of the Child Health for Madhya Pradesh, Additional Mission and Director of the Education Department)
February 2017	Resubmission of protocol and short report to LVPEI and LSHTM Ethics Committees and Madhya Pradesh government
March to May 2017	Village consent, village mapping and piloting of enumeration forms
July 2017 to January 2018	Enumeration of the participants and data entry
September 2018 to April 2019	Indian Council of Medical Research (ICMR) application and approval
November 2018 to January 2019	Knowledge Attitudes and Practice (KAP) survey interviews and data entry
April to June 2019	Catch-up enumeration
19 June 2019	Randomisation
June to December 2019	Intervention design, village sensitisation and trainings
October 2019	STRIPES2 intervention starts
December 2019	CHAMPION2 intervention starts
19 June 2020	Neonatal and maternal survival start to count to final analysis
August 2020	First survey of children’s enrolment and attendance in school
February 2021	Second survey of children’s enrolment and attendance in school
March/April 2021	Final numeracy and literacy tests for children (STRIPES2)
November 2021	Statistical analysis of STRIPES2 completed
December 2022	Final data collection for CHAMPION2
May 2023	Statistical analysis of CHAMPION2 completed

### Sample size {14}

The process by which clusters (villages) were selected is described subsequently (see “Recruitment”). After application of the first three criteria to identify clusters (steps 1–3), there are 484 villages that are potentially eligible for the trial. Originally it had been the intention to randomise 300 villages, because this gave over 90% statistical power to detect (1) a 20% reduction in neonatal mortality in CHAMPION2 and (2) a difference of 0.25 SD in mean standardised test scores in STRIPES2. However, incorporating the buffer zones described in the village selection procedure as described previously meant that only 204 villages could be selected. These 204 villages have a mean population of 1487 (minimum 558, maximum 2490) and SD of 505 (equating to a coefficient of variation of 0.34). Estimating the number of children in each school year from the number younger than 6 years (divided by 6), the mean number of children in each school year is 38.3 (minimum 20, maximum 71) with SD of 13.3 (coefficient of variation 0.35). Assuming that 25% of the children will not be eligible according to the criteria, this gives an estimated mean number of eligible children per village of 28.7 with a minimum of 15.

In CHAMPION the design effect for neonatal mortality was 1.306, equating to an intra-cluster correlation coefficient (ICC) of 0.011 (with allowance for variability in cluster size, the assumed coefficient of variation = 0.34). For CHAMPION2, allowing for the fact that each village has an average population of 1487 and estimated crude birth rate of 30.7 per 1000 population per year in rural areas of Satna district [3], 114 births per village over a 30-month follow-up period are expected. Assuming (1) an ICC of 0.011 for the primary outcome, (2) an assumed coefficient of variation for village size variability of 0.34, (3) that 5% of villages will be excluded for reasons such as withholding consent, and (4) that there will be 10% loss to follow up, a trial with 194 villages (95% of 204) has 75% power (5% two-sided significance) to detect a 20% reduction in neonatal mortality from 6.7% to 5.36% and 91% power (5% 2-sided significance) to detect a 25% reduction in neonatal mortality from 6.7% to 5.0% (this and other power and sample size calculations were performed using the “power” command in Stata 14) [43]. Since the reduction in neonatal mortality seen in CHAMPION was 25%, proceeding with 204 villages seems reasonable given the requirement for buffer zones in order to avoid contamination.

We estimated that the 204 villages will include an average of 28.7 eligible students (5740 students in total). In the STRIPES trial the estimated effect was a 0.75 SD increase in mean score: however, effects of smaller magnitude than this would still be important to detect. Conservatively assuming that 60% of the eligible children (4860 students) will take the test at the end of the trial and an ICC of 0.23 (as seen in the STRIPES trial) then a trial with 194 villages (i.e. assuming that 5% of the 204 villages will not take part) will give 88% power to detect a difference of 0.25 SD in mean standardised scores between intervention and control villages using a conventional two-sided significance level of 5% (assuming a coefficient of variation of 0.35 in numbers taking the test by village). If the treatment effect is of the order of that seen in the STRIPES trial then there will be reasonable statistical power to explore interactions by ethnicity, gender, wealth, and geographic location.

As described above, in the sample size calculation we anticipated that 194 of the 204 villages would be randomised. In fact, 196 were randomised, with 6 villages removed since they were found to be too close to urban areas to be considered rural, and 2 removed because insufficient eligible children were found.

### Recruitment {15}

Following discussion with the *Sarpanch* (the head of a *Panchayat*, which is a group of villages), all eligible children and women were enumerated in all villages in which we obtained village consent. These villages were selected from all villages in Satna district that satisfy the eligibility criteria.

#### Cluster identification

The pathway to select villages (clusters) was the following:

1. We selected Satna district from the census 2011 data;
2. The *tehsils* of Birsinghpur, Majhgawan and Raghurajnagar were excluded (difficult access, risk of violent robbery, and urban sub-district);
3. Villages with a population larger than 2500 and fewer than 120 children under the age of 6 years (suggesting fewer than 20 children in each school year) were excluded;
4. Villages within 5 km of a CHC or CH were excluded;
5. Using a programme developed in the statistical package Stata, villages were selected using an algorithm that attempts to maximise both (i) the total number of villages selected and (ii) the population of selected villages, whilst ensuring that there is a distance of at least 3 km between the centre of each pair of selected villages (buffer zones to reduce contamination);
6. During the enumeration period, we will identify the eligible children, women and men in each selected village;
7. If a selected village cannot be included (if, for example, consent is refused or there are insufficient eligible children) then this village should be dropped from the selected list and can be replaced by one or more substitute villages not originally selected, provided that there is a distance of at least 3 km between each pair of selected villages.

#### Randomisation

Randomisation was at the village (cluster) level, with stratification by village size and distance to the nearest CHC or CH.

#### Control groups

CHAMPION2 control villages will receive the STRIPES2 intervention and the usual health services provided by the government, private, and other Non-Governmental Organisations (NGOs). STRIPES2 control villages will receive the CHAMPION2 intervention and the usual education services provided by the government and other NGOs.

It is anticipated that the CHAMPION2 intervention will have negligible impact on children’s learning scores, and the STRIPES2 intervention will have negligible impact on neonatal mortality. The fact that these interventions are being carried out may improve the extent and quality of the data collected in the control villages. The relevant interventions will be expanded into the controls if proven to be successful.

#### Randomisation flowchart

**Fig. 1:**
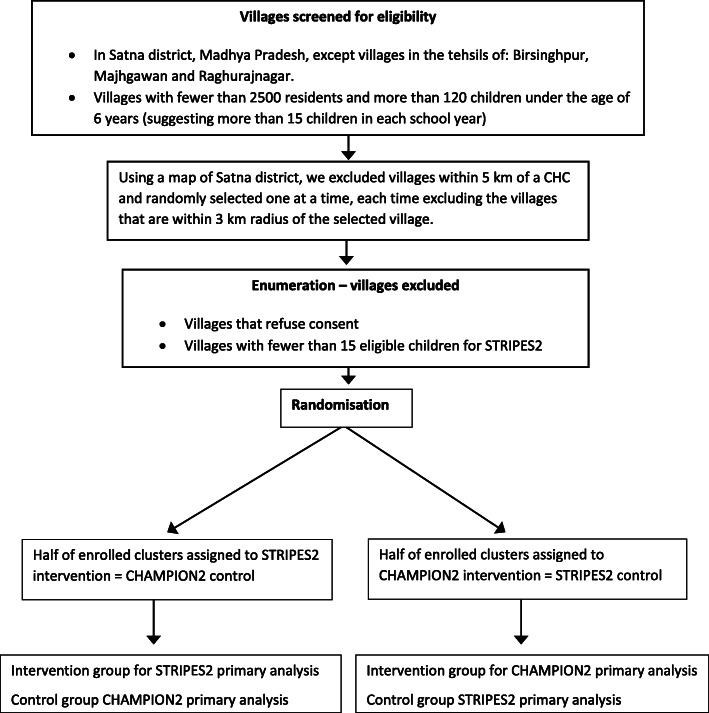
Flowchart of villages through trial. CHC, Community Health Centre

The process from village selection to inclusion in the final analysis is shown in Fig. 1.

## Assignment of interventions

### Sequence generation {16a}

The trial statistician based at the London School of Hygiene and Tropical Medicine randomised the clusters using a random number generator. Randomisation was stratified by population size and distance to the nearest CHC or CH.

### Concealment mechanism {16b}

All villages were simultaneously randomised on 19 June 2019 using a random number generator. Following randomisation (which was post enumeration and cluster consent), the trial statistician informed the intervention providers (NICE and Pratham) about which villages had been allocated to each intervention. Since there was no sequential allocation there was no need for a concealment mechanism after the initial randomisation. The intervention providers then informed the villages. Allocation was concealed up to that point.

### Implementation {16c}

Independent teams hired and trained by GH Training and Consulting registered the participants. The trial statistician at the London School of Hygiene and Tropical Medicine performed randomisation, and then shared the allocation information with the intervention providers. Once the lists of villages allocated to receive the health and education packages were shared with NICE and Pratham respectively, each intervention provider started the process of community sensitisation.

## Assignment of interventions: blinding

### Who will be blinded {17a}

Due to the nature of the interventions, it will not be possible to have a blinded study. Participants will be aware as to whether they are in the CHAMPION2 or STRIPES2 intervention arm. Nevertheless, all physicians who will be assigning a cause of death using the World Health Organisation’s verbal autopsy toolkit [44] in CHAMPION2, and the teams who will be carrying out testing in STRIPES2 (hired and trained by GH Training and Consulting)^2^ will be blind to the randomisation.

### Procedure for unblinding if needed {17b}

The trial is not blinded, so there is no need for unblinding.

## Data collection and management

### Plans for assessment and collection of outcomes {18a}

An independent research team based in Satna will manage the enumeration, monitoring, surveys and tests. Except for the list of eligible participants in the trial, no further data will be shared by the research team during the trial. A set of procedures will be established to recruit, train and monitor the research staff, preserving the segregation from the intervention group.

It will be possible to locate enumerated participants including women, unmarried men, children, and parents, by assigning an ID number that is a combination of the village identifier, and household identifier.

For CHAMPION2, women were interviewed during enumeration about their previous obstetric and pregnancy-related history. Neonatal deaths were assessed for the year before enumeration through recall based on a truncated pregnancy history. Once the intervention starts, participating women will be continuously monitored. Unmarried men will also be enumerated and monitored to determine if they marry. If an unmarried man marries, his wife becomes a resident of the village, and if she gives consent to participate in the trial, then she will be followed up in cycles of 30–33 days.

Research teams will visit all the trial villages in a cycle of 30–33 days to register new pregnancies, follow up those already recorded, interview for pregnancy outcomes and follow up the neonatal status until the baby is age 29 days. Research teams will also monitor enumerated unmarried men to determine if they have recently been married. If a woman is recorded to be pregnant and leaves the village, the research team should attempt to ascertain her pregnancy outcome from her as part of the cycle of household visits, or by calling her over the phone or interviewing her family (if she does not come back to the village).

Deaths (neonates up to and including the 28th day of life or women who died pregnant or within 42 days after the delivery) will be investigated to ascertain the possible cause of death using verbal autopsies developed by the WHO and adapted for local understanding. Stillbirths will be defined as the death of a foetus from the 28th week of gestation and before birth [45]. To ascertain the weeks of gestation we will use the last menstrual period (LMP) recorded when research teams first become aware that a woman is pregnant. Two physicians, blinded to allocation, will independently assign the cause of death. In the case of disagreement on one main cause of death, a third physician will be recruited to resolve the disagreement.

In STRIPES2, a follow-up survey will be conducted twice during the trial to update the status (residence, school enrolment, and attendance) of those children enlisted. In one of these surveys, we will also record information to generate a wealth index.

Literacy and numeracy tests will be administered by the end of the intervention to all children enumerated who are available in the village on the test day. Both the tests will be designed to measure a student’s foundation skills in literacy and numeracy in the early grades.

### Plans to promote participant retention and complete follow up {18b}

There will be no rewards or incentive for participants, except that children will be offered a small gift when taking the final test (such as a snack or piece of stationery, which will be decided upon close to the date of the test). All the outcome data that has been collected up to the last monitoring for CHAMPION2 and in the final test for STRIPES2 will be included in the analysis unless consent has been withdrawn.

### Data management {19}

All data related to pregnancies, births, deaths, children’s school enrolment, parent’s engagement in the child’s education and the final EGRA and EGMA (literacy and numeracy) tests will be double-entered in the main office of the research team in Satna. The database has been developed by Sealed Envelope (https://www.sealedenvelope.com), an independent company contracted to construct and maintain a bespoke database for the trial, who will also keep a periodical backup of the data. Paper forms will be stored there in a secure location and destroyed after the statutory period expires.

### Confidentiality {27}

All data will be kept strictly confidential - names will be removed from the database before analysis and the paper data collection instruments will be kept in a secure location in Satna, Madhya Pradesh, and destroyed after the statutory period expires.

## Statistical methods

### Statistical methods for primary and secondary outcomes {20a}

Prior to commencing statistical analysis, a detailed statistical analysis plan will be written by the trial statistician. This will be considered by the trial Steering and Data Monitoring Committees and signed off by the Principal Investigator before statistical analysis is carried out.

#### CHAMPION2

The primary analysis of all outcomes will follow the intention-to-treat principle, with a secondary per-protocol analysis of the primary outcome also undertaken. As this trial has a complex hierarchical structure, with multiple women per cluster, potentially multiple pregnancies per women, and potentially multiple births per pregnancy, we will use a generalised estimating equations (GEE) analysis approach [46]. This assumes non-independence of all observations from the same cluster, and accounts for non-independence of multiple outcomes from the same woman.

For the primary outcome, the relative risk with a 95% confidence interval will be obtained from a GEE model with a binary outcome, a log link, a “working” assumption of independence with robust standard errors to take account of clustering. The model will include the stratifying variables [47, 48]. The risk difference with a 95% confidence interval will be obtained from a GEE model with a binary outcome, an identity link, a “working” assumption of independence with robust standard errors to take account of clustering. This model will also include the stratifying variables. The numbers of lives saved will be estimated by multiplying the number of live births in the intervention arm by the estimated risk difference. Relative risks will be estimated for secondary binary outcomes, using the same approach as for the primary outcome.

#### STRIPES2

The primary analysis of all outcomes will also follow the intention-to-treat principle, with a secondary per-protocol analysis of the primary outcome also undertaken. Mean child-specific composite test scores at the end of the final academic year will be compared using regression models with adjustment for stratification factors and with robust standard errors to allow for clustering by village. Bootstrap confidence intervals will be reported for non-normally distributed continuous outcomes. Intervention–gender interactions will be tested.

A secondary per-protocol analysis of the respective primary outcomes will be carried out in both CHAMPION2 and STRIPES2. This will exclude participants in the respective intervention arms deemed not to have satisfied adherence criteria that will be pre-specified in the statistical analysis plan.

#### Economics

We will measure the cost effectiveness from the provider point of view. Project cost will be collected prospectively and converted to economic cost. Cost data will be adjusted for inflation, using the Indian consumer price index and reported in Indian Rupees and US dollars in the equivalent period. Incremental cost effectiveness will be measured relative to the status quo alternative as defined by outcomes in the control arms.

### Interim analyses {21b}

CHAMPION2 interim analyses will be pre-specified and provided confidentially by the trial statisticians to an independent Data Monitoring Committee (DMC), which will be guided by the Peto-Haybittle rule [49] with a recommendation to stop made on the basis of a large and statistically significant (*p* < 0.001) difference in either direction. The DMC will report to the Trial Steering Committee (TSC). As in STRIPES2 there are no concerns about the safety of the intervention, and as there is only one assessment of the primary efficacy outcome, there will be no such interim analysis for STRIPES2.

### Methods for additional analysis {20b}

Secondary analyses in both CHAMPION2 and STRIPES2 will extend the models described above for the respective primary outcomes to (separately) investigate interactions by the two stratification factors (village size and distance to the nearest Community Health Centre or Civil Hospital).

### Methods in analysis to handle protocol non-adherence and missing data {20c}

Per-protocol analysis will be carried out in both CHAMPION2 and STRIPES2. This will exclude participants in the respective intervention arms deemed not to have satisfied adherence criteria that will be pre-specified in the statistical analysis plan. In the event of substantial missing data, secondary analysis using techniques such as multiple imputation will be considered, with details pre-specified in a supplementary statistical analysis plan.

### Plans to give access to the full protocol, participant level-data, and statistical code {31c}

The protocol will be publicly available from this website. The datasets analysed during the current study are available from the corresponding author on reasonable request.

## Oversight and monitoring

### Composition of the coordinating centre and trial steering committee {5d}

#### Trial management group

The trial will have independent implementation and research teams. A TSC, with independent membership (an obstetrician, a paediatrician, and an education researcher) will supervise the trial.

#### Implementation team

The team in charge of implementing and managing the CHAMPION2 intervention will be composed of:

1. The Programme Officer (PO) who will oversee the implementation of the whole programme assisted by junior programme officers (JPOs);
2. ASHAs (VHWs) who will be responsible for the following: mobilising the community for FDS, facilitating the community mobilisation with women’s groups, monitoring pregnant women, imparting health education, helping to conduct home deliveries in emergency situations, and facilitating referrals;
3. Nurse midwives who will: provide services in the FDS, supervise the ASHAs (VHWs), help deliver babies if needed, and give logistical support in the villages;
4. Field Monitors who will supervise ASHAs (VHWs), facilitate FDS, and monitor referrals to health facilities;
5. Consultants in maternal and neonatal health, community mobilisation and support, and liaison who will monitor patient care logistics and outcomes, build team capacity in community sensitisation, and support in resolving community-level issues;
6. A team lead by JPO will be conducting and managing the Participatory Learning and Action (PLA).

The STRIPES2 intervention team will consist of:

1. National and state team members who will provide ongoing guidance and support to the programme;
2. A Programme Head (PH) who will liaise with the local government officers and with internal state and central teams. She/he will oversee the teams’ operations on the ground and be responsible for implementation of the education intervention;
3. Two Content Associates who will provide content support to the teams. They will work with members from Pratham’s central content team to design/develop teaching and learning materials for the intervention;
4. A Monitoring, Measurement and Evaluation Associate who will be responsible for training teams on and analysing internal measurements;
5. Cluster leaders (CLs) - each CL will be responsible for about 10 villages. They will provide training and supervision to the Pratham instructors, whilst also facilitating engagement with the community;
6. Pratham instructors (PIs) who will lead the classes, monitor every child’s learning progress, and liaise with mothers.

The research team responsible for enumerating all the participants, monitoring pregnant women and neonates, conducting the tests, and collecting the data that will be used to evaluate the impact of the interventions is composed of:

1. One Project Coordinator who will be responsible for leading the research teams (health and education), organising training, and liaising with other Coordinators and the Database team;
2. Survey Enumerators who will visit households and conduct interviews during enumeration and for the annual survey (STRIPES2);
3. Village Enumerators (VEs) who will be monitoring whether the enumerated women are alive, pregnant, and present in the village during the 30–33-day cycle (CHAMPION2);
4. Data Supervisors (DSs) who will lead the field research teams and supervise and facilitate the work of all enumerators. They will verify the completed forms and conduct quality checks to validate them; monitor neonates; and complete pregnancy outcome questionnaires, verbal autopsies and interviews to enrol women married into the trial villages;
5. A Training Coordinator who will organise induction and team monthly meetings, and liaise with the Database Manager and field teams for data-related issues;
6. Data Supervisors for Verbal Autopsies (DSVA) who are responsible for conducting the verbal autopsy interviews (MVA and NVA);
7. Cluster Coordinators (CC) who will *c*oordinate the work of the data supervisors; lead meetings; organise the distribution of forms; and engage in community interaction along with supervisors;
8. A Field Manager who will be in charge of the monitoring-cycle data (CHAMPION2). She/he will select the field staff, organise the forms distribution, and verify the quality and validate the data collected;
9. A Database Manager who will be responsible for coordinating the data entry operators; correcting double entry inconsistencies; coordinating the printing and distribution of forms; liaising with the database programmer in London; and manage the database and assure its integrity;
10. Data Entry Operators who will double-enter all the data;
11. Physicians who will attribute the cause of death at maternal and neonatal verbal autopsies;
12. Test Administrators who will conduct the final tests for STRIPES’ evaluation.

### Composition of the Data Monitoring Committee (DMC), its role and reporting structure {21a}

The CHAMPION2 DMC includes a statistician and a clinician, both independent of the trial and the Sponsor. The primary role of the DMC is to safeguard the interests of the study participants and to enhance the integrity and credibility of the trial. The DMC will report to the TSC.

### Adverse event reporting and harms {22}

Information will be collected about harms - serious adverse events (SAEs). An SAE is formally defined as any untoward occurrence that results in death; is life-threatening; requires inpatient hospitalisation or prolongation of existing hospitalisation; results in persistent or significant disability/incapacity; or is a congenital anomaly/birth defect [50].

For this trial, SAEs are defined as deaths (stillbirths, neonatal, and maternal), maternal blood transfusions, hospital admissions other than for labour, and prolongation of existing hospitalisation (beyond 48 h). Information about persistent or significant disability/incapacity or congenital anomaly/birth defect will not be collected due to the difficulty of ascertaining this in routine data collection in this trial but if a child dies, information about birth defects will be collected within the verbal autopsy process. Information about SAEs will be collected as part of routine outcome data collection in the villages by the research team.

In addition, if any other serious but unexpected adverse event is seen which might be related to a trial intervention, this should be logged by calling the trial coordinator (TC) and a written SAE report submitted. The TC will coordinate the reporting of all such events within 7 days to the L V PRASAD EYE Institute and London School of Hygiene and Tropical Medicine Ethics Committees. This expedited reporting will be limited to those outcomes not already listed as primary or secondary outcomes, yet which might reasonably occur as a consequence of the trial intervention.

### Frequency and plans for auditing trial conduct {23}

The TMG will meet (remotely) approximately once a month. The TSC and the independent DMC and Ethics Committee will meet approximately annually.

### Plans for communicating important protocol amendments to relevant parties (e.g. trial participants, ethical committees) {25}

The TMG will notify relevant partners of changes to the protocol and update the clinical trial registry. The DMC will be informed about any major deviations from protocol. We will provide updates of the protocol in the clinical trial registry and inform the Ethics Committees of major changes.

## Dissemination plans {31a}

We will attempt to disseminate the results of the trial in international, national, state and district conferences, and to villages participating in the trial, and publish the findings in peer-reviewed journals following the Consolidated Standards for Reporting Trials (CONSORT) guidance for cRCTs [51]. All publications must be approved by the Trial Steering Committee.

## Trial status

MP trial protocol Version 11th Version - 20 March 2020.

Recruitment of participants started on 1 June 2017 and will continue till approximately December 2021 for eligible women (CHAMPION2). For STRIPES2, no more children will be added to the list of participants.

Randomisation was in June 2019. The trial interventions are scheduled to start from October–December 2019.

### Definitions

Early neonatal deaths: deaths of neonates within 7 days of delivery (i.e. 0–7 days) [39].

Emergency Obstetric Care (EmOC): a package of medical interventions required to treat seven obstetric complications [34]. The medical interventions include parenteral antibiotics, oxytocic drugs and anticonvulsants, manual removal of placenta, removal of retained products, and assisted vaginal deliveries [35].

Late neonatal deaths: neonatal deaths occurring between the 8th and 29th day of life [39].

Maternal death: defined by the World Health Organisation as death of a women whilst pregnant or within 42 days of termination of pregnancy, irrespective of the duration and site of pregnancy, from any cause related to or aggravated by the pregnancy or its management but not from accidental or incidental causes [39].

Maternal mortality ratio: the ratio of maternal deaths per 100,000 live births [52].

Neonatal mortality rate: the number of deaths of a new born infant during the first 28 completed days of life per 1000 live births [39].

Perinatal deaths: refers to pregnancy loss at or beyond 28 weeks gestation and early neonatal dates within 7 days of delivery [39].

Perinatal mortality rate: the number of perinatal deaths per 1000 total births in a given year. The perinatal period starts at the beginning of foetal viability (28 weeks of gestation) and ends at the end of the 7th completed day after delivery. Perinatal deaths are the sum of stillbirths plus early neonatal deaths [41].

*Sarpanch*: an elected head of a village-level statutory institution of local self-government called the *Panchayat* (village government) in India, Pakistan, and Bangladesh. The *Sarpanch* is the focal point of contact between government officers and the village community.

Stillbirth: refers to pregnancy loss at or beyond 28 weeks gestation without any signs of life [41].

## Abbreviations

ANC: Antenatal care
ASCs: Academic support classes
ASER: Annual Status of Education Report
ASHA: Accredited Social Health Activist
CH: Civil Hospital
CHC: Community Health Centre
CL: Cluster Leader
CRA: Cooperative reflective approach
cRCT: Cluster randomised controlled trial
CE: Community Educator
DMC: Data Monitoring Committee
EDD: Expected delivery date
EGRA: Early Grade Reading Assessment
EGMA: Early Grade Mathematics Assessment
EI: Effective intervention
EmOC: Emergency Obstetric Care
FDS: Fixed Day Services
GCP: Good Clinical Practice
GEE: Generalised estimating equations
ICC: Intra-cluster correlation coefficient
ICH: International Conference on Harmonisation
ICMR: Indian Medical Council of Research
IMNCI: India’s Integrated Management of Childhood Illness
IPHS: Indian Public Health Standards
JE: Janani Surakhsa Express
KAP: Knowledge attitudes practice
LMP: Last menstrual period
LSHTM: London School of Hygiene and Tropical Medicine
LVPEI: L V PRASAD EYE Institute
NCERT: National Council of Educational Research and Training
NGO: Non-Governmental Organisation
NMR: Neonatal mortality rate
NMW: Nurse Midwife
PI: Pratham Instructor
PLA: Participatory learning and action
PNC: Postnatal care
SCERT: State Council Educational Research and Training
SNCU: Special Newborn Care Unit
TSC: Trial Steering Committee
VHW: Village Health Worker
WHO: World Health Organisation

## Footnotes

1 Children were included in cases where the day of birth was missing at enumeration but the child was born between June 2010 and June 2013.

2 The final literacy and numeracy tests will be administered by a person blind to which villages are holding the Pratham classes.
